# Exosomal MicroRNA as Biomarkers for Diagnosing or Monitoring the Progression of Ovarian Clear Cell Carcinoma: A Pilot Study

**DOI:** 10.3390/molecules27123953

**Published:** 2022-06-20

**Authors:** Kayo Horie, Naoki Nanashima, Yoshihito Yokoyama, Haruhiko Yoshioka, Jun Watanabe

**Affiliations:** 1 Department of Bioscience and Laboratory Medicine, Hirosaki University Graduate School of Health Sciences, Hirosaki 036-8564, Japan; n_nanashima@ms.auhw.ac.jp (N.N.); yoshioka@hirosaki-u.ac.jp (H.Y.); watajun@hirosaki-u.ac.jp (J.W.); 2 Department of Obstetrics and Gynecology, Hirosaki University Graduate School of Medicine, Hirosaki 036-8203, Japan; yokoyama@hirosaki-u.ac.jp

**Keywords:** exosome, microRNA, ovarian cancer, clear cell carcinoma of the ovary, tumor markers

## Abstract

Ovarian cancer is the most common cause of gynecological malignancy-related mortality since early-stage disease is difficult to diagnose. Advanced clear cell carcinoma of the ovary (CCCO) has dismal prognosis, and its incidence has been increasing in Japan, emphasizing the need for highly sensitive diagnostic and prognostic CCCO biomarkers. Exosomal microRNAs (miRNAs) secreted by tumor cells are known to play a role in carcinogenesis; however, their involvement in ovarian cancer is unclear. In this study, we performed expression profiling of miRNAs from exosomes released by five cell lines representing different histological types of ovarian cancer. Exosomes isolated from culture media of cancer and normal cells were compared for miRNA composition using human miRNA microarray. We detected 143 exosomal miRNAs, whose expression was ≥1.5-fold higher in ovarian cancer cells than in the control. Among them, 28 miRNAs were upregulated in cells of all histological ovarian cancer types compared to control, and three were upregulated in CCCO cells compared to other types. Functional analyses indicated that miR-21 overexpressed in CCCO cells targeted tumor suppressor genes *PTEN*, *TPM1*, *PDCD4*, and *MASP1*. The identified miRNAs could represent novel candidate biomarkers to diagnose or monitor progression of ovarian cancer, particularly CCCO.

## 1. Introduction

Ovarian cancer is the most common cause of death from gynecological malignancies [1], and epithelial ovarian cancer (EOC) is the most prevalent type, accounting for almost 90% of ovarian cancer cases. The poor prognosis of EOC is attributed to the disease being typically diagnosed at an advanced stage due to the lack of early screening [2,3]. The fifth edition of the 2020 World Health Organization classification identifies at least five main subtypes of ovarian carcinoma based on histopathology, immune profile, and molecular analysis: high-grade serous, low-grade serous, endometrioid, clear cell, and mucinous carcinomas [3]. 

Clear cell carcinoma of the ovary (CCCO) is a distinct EOC type in terms of clinical, histopathological, and genetic features. Overall, CCCO prognosis is good, since many patients are diagnosed at stage I. However, advanced CCCO has dismal prognosis because of its resistance to standard treatment, which is also characteristic for recurrent disease [4,5]. CCCO mostly occurs on the background of endometriosis, and many cases with mutations in *ARID1A* and *PIK3CA* genes have been observed [6,7]. The global CCCO incidence rate is low; however, it significantly differs among ethnic groups [8]. Thus, CCCO is rather rare in western countries but is not uncommon in Asia [4], particularly in Japan, where the incidence rate of CCCO is increasing; it currently exceeds 25% [5]. Therefore, the discovery of highly sensitive diagnostic and prognostic biomarkers of CCCO is urgent for Japan.

Exosomes are vesicles with diameters of 30–200 nm [9,10]. They are secreted through endocytosis by most cell types, including cancer cells and contain proteins, lipids, DNA, mRNA, microRNA (miRNA), and other molecules [11,12,13]. Recent advances in exosome research have further improved our understanding of the biosynthesis and uptake of exosomes [9]. MiRNAs are small regulatory non-coding RNAs of approximately 22 nucleotides and are known to regulate gene expression by binding to the 3′ untranslated region (3′-UTR) of the target RNA in a sequence-complementary manner [14]. Furthermore, it has become clear that the binding site of miRNA also exists in the 5′UTR of the target RNA and the protein coding region [15,16]. MiRNAs play important roles in various physiological and pathological processes [14], including cancer, and their value as potential cancer biomarkers has been acknowledged [17]. 

Lötvall et al. [12] have reported that mRNAs and miRNAs can be delivered by exosomes to other cells and function in the new location. Exosomal miRNAs are involved in many biological processes, including cancer development and progression. They are protected from enzymatic degradation and are relatively stable in body fluids [17,18,19]. Thus, exosomes represent excellent delivery vehicles of functionally active miRNAs.

At present, carbohydrate antigen 125 (CA125) is the most widely used biomarker for screening ovarian cancer; however, it is not accurate enough for diagnosing early-stage disease [20], since CA125 serum levels may also increase under normal physiological conditions such as menstruation and by non-cancerous cell growth such as uterine fibrosis [21]. Therefore, it is necessary to develop methods of early diagnosis and identify new molecular biomarkers for ovarian cancer. Recently, it has been suggested that exosomes can be used in ovarian cancer diagnostics. Exosomes derived from ovarian cancer cells have been reported to carry about 2000 proteins and are detected in various body fluids such as ascitic fluid and peripheral blood [22]. 

Analysis of exosomal miRNAs in ovarian cancer is usually performed using plasma samples [23,24]. However, since exosomes are released into body fluids from various types of cells, it is difficult to detect miRNAs specifically expressed in cancer cells. Furthermore, EOC subtypes may behave as distinct diseases because their cellular origin, histology, and treatment responses differ. Therefore, identifying EOC subtype-specific biomarkers for diagnosing early-stage disease would be clinically beneficial [25].

In this study, we performed profiling of exosomal miRNAs expressed in various histological types of ovarian cancer using specific cell lines. To the best of our knowledge, this is the first study to compare exosomal miRNAs in various EOC histological subtypes. We also detected three exosomal miRNAs that were overexpressed in CCCO than in other histological types. Although the results of the current study should be validated in clinical samples, they reveal novel potential diagnostic and/or prognostic biomarkers of EOCs and CCCO.

## 2. Results and Discussion 

### 2.1. Nanoparticle Tracking Analysis of Exosomes Isolated from Cell Culture Media

**Figure 1:**
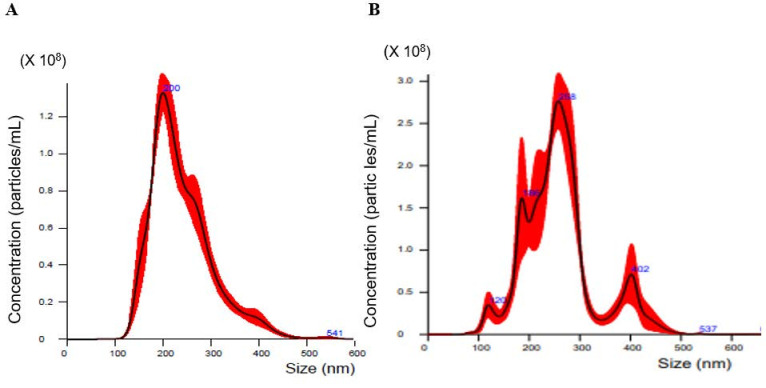
Nanoparticle tracking analysis (NTA) of extracellular vesicles (EVs) from ovarian cancer cell culture media. Representative particle size distribution profiles for MCAS (**A**) and HAC-2 (**B**) cells are shown.

In this study, we investigated cell lines representing the five main subtypes of ovarian carcinoma: serous (HRA), endometrioid (TOV-112D), clear cell (HAC-2 and OVAS), and mucinous (MCAS). The human ovarian surface epithelial (HOSE) cell line was used as a control. Nanoparticle tracking analysis (NTA) is commonly applied to determine the concentration and diameter of extracellular vesicles (EVs), including exosomes [26]. The size distribution of exosomes secreted by ovarian cancer cells into the culture medium was analyzed with NanoSight; representative profiles are shown in Figure 1. High concentrations of 100–400 nm particles were detected in culture media of both MCAS (Figure 1A) and HAC-2 (Figure 1B) cells; the mode diameter value was approximately 200 nm.

Table 1 lists the concentration and diameter of EVs isolated from culture media of ovarian cancer cell lines representing different histological subtypes. The mean diameter was 120–260 nm, while the mode value was 89–215 nm.

Recent reports have classified EVs into the following three groups based on size and biogenesis: exosomes (30–200 nm), microvesicles (100–1000 nm), and apoptotic bodies (>1000 nm) [9,10]. In this study, we focused on exosomes. There was considerable variation in EV concentrations among the cell lines; thus, the highest and lowest concentrations, which were observed in MCAS and HRA cell media, respectively, differed widely, where the highest was 40 times the lowest concentration. The content and function of exosomes depend on the cell source and conditions under which they are produced [27]. To the best of our knowledge, there have been no studies on quantitative comparison of exosome release in different cancer cells. At present, it is clearly understood that mucinous ovarian carcinoma differs from all other EOCs [28]. This is consistent with our results which show significantly higher EV release by MCAS cells compared to that released by other cell lines. Further experiments are required to confirm this finding. 

### 2.2. Characterization of Exosomes by CD63 Expression

**Figure 2:**
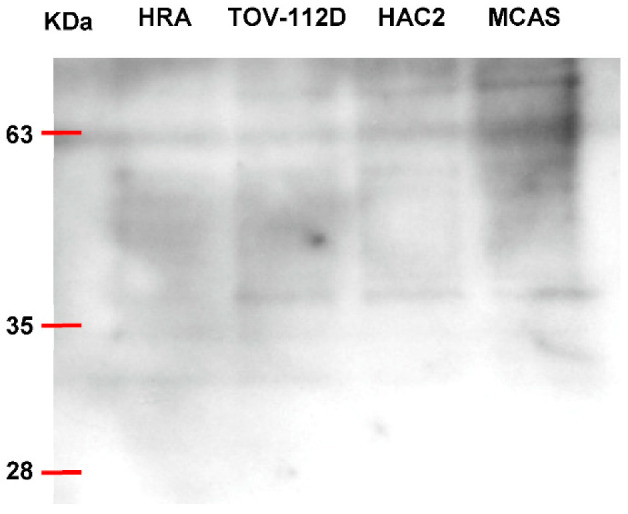
Exosome detection based on the expression of exosomal marker CD63. Exosomes isolated from culture media of the indicated ovarian cancer cell lines were subjected to western blotting analysis using anti-CD63 antibody.

We further confirmed the presence of exosomes by Western blotting analysis (Figure 2). Several studies have shown that vesicles with features of exosomes are enriched in tetraspanins such as CD63 and CD81, which are used as specific exosomal markers [22,29]. 

All exosome preparations showed a smeared CD63-specific band of 30–60 kDa. The culture medium of MCAS cells showed the strongest CD63 signal, which was consistent with the NanoSight results, suggesting that MCAS cells released the largest number of exosomes.

### 2.3. Validation of Small RNA Quality and Concentration by Electrophoresis

**Figure 3:**
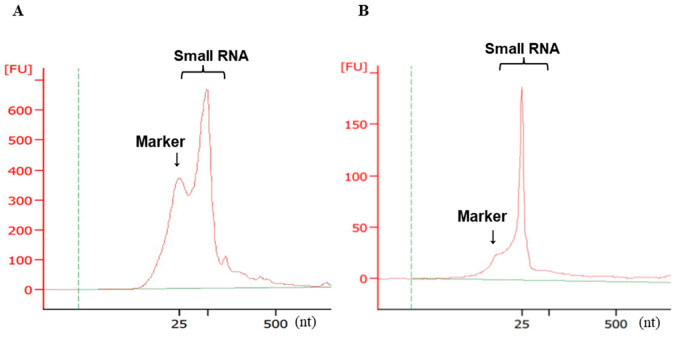
Representative images of the densitometry profiles of miRNAs isolated from exosomes. (**A**) MCAS, (**B**) HAC-2 cells. The vertical and horizontal axes indicate fluorescence units (FU) and RNA length in nucleotides (nt). The peak for small RNAs (25–200 nt) corresponds to exosomal miRNAs; marker indicates the internal standard (25 nt).

Figure 3 shows representative densitometry results for isolated miRNAs. Quantitative and quality checks of RNA using Bioanalyzer revealed a high peak in the miRNA area; ribosomal (r)RNA with 28S and 18S peaks at the 2:1 ratio was not detected. These results are agreement with a report that exosomes are rich in small RNAs but do not have prominent rRNA peaks [30]. 

### 2.4. Microarray Expression Profiling of Exosomal miRNAs from Ovarian Cancer Cells Compared to Normal Cells

**Figure 4:**
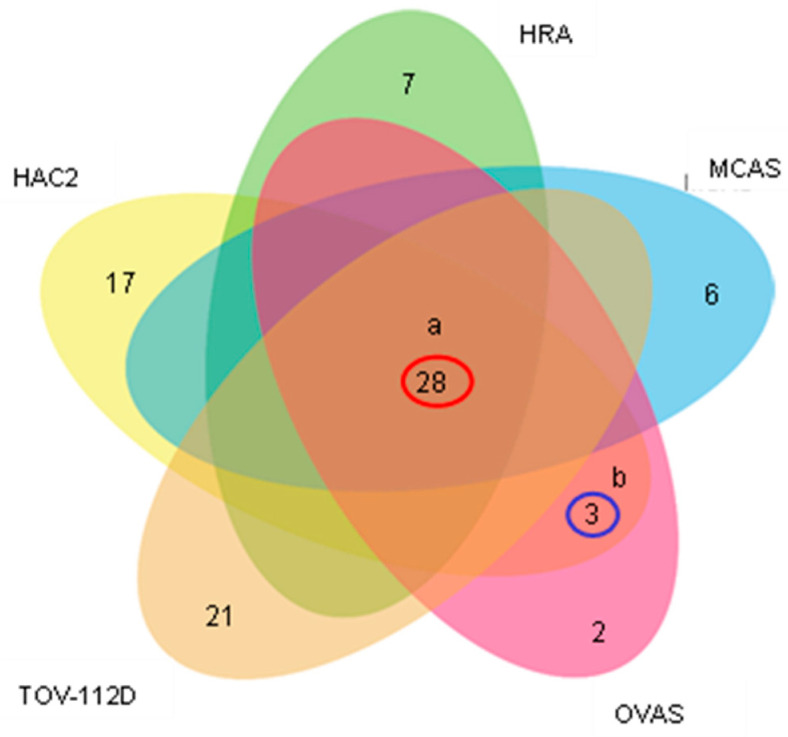
Upregulation of exosomal miRNAs in cell lines representing different histological ovarian cancer subtypes compared to normal cells. The Venn diagram was constructed based on microarray analysis. The numbers of miRNAs overexpressed in each cell type are indicated. The numbers of exosomal miRNAs commonly upregulated in all cell lines compared to control (a) and specifically upregulated in clear cell carcinoma compared to the other histological types (b) are circled red and blue, respectively.

Next, we compared exosomal miRNA levels in ovarian cancer cells with that in normal cells using microarrays. In cancer cells, the expression of 143 miRNAs was increased by ≥1.5-fold compared to that in control cells. The cut-off difference of 1.5-fold expression is based on several miRNA profiling studies, confirming the subtle changes in miRNA expression [31,32,33,34,35]. A Venn diagram shows the numbers of upregulated miRNAs in each cell line; those overlapping in all ovarian cancer subtypes are circled in red (Figure 4). These results indicate that 28 exosomal miRNAs could be used as biomarkers for the early diagnosis of EOC. In addition, we assessed the similarities and differences between histological subtypes (Table S1).

### 2.5. Analysis of Exosomal miRNAs Overexpressed in All Ovarian Cancer Histological Subtypes 

Table 2 lists the 28 miRNAs that showed increased expression in all cells representing the different histological ovarian cancer types compared to those in normal HOSE cells. Our results are consistent with previous studies showing that exosomal miRNA expression profiles in the plasma or serum of patients with ovarian cancer significantly differs from those of patients with benign disorders [36]. Resnick et al. [37] have reported that serum levels of miR-21, -29a, -92, and -93 were significantly higher in patients with ovarian cancer than those in healthy individuals. Our results also indicated that miR-21 was strongly expressed in CCCO but not in other histological subtypes; however, it can be different in serum samples and culture media. Another study has shown that miR-93-5p plasma levels are significantly increased in patients with ovarian cancer compared to those in controls [23]; furthermore, miR-93 is overexpressed in ovarian cancer cell lines resistant to cisplatin [38,39]. 

miR-1237 is significantly associated with survival of patients with ovarian cancer [40]. Also, miR-320 is strongly downregulated in EOC tissues and cell lines and is significantly correlated with cancer stage and lymph node metastasis in patients with EOC [41]. The levels of these miRNAs were increased in the exosomes of ovarian cancer cell lines in our study (Table 2). However, it should be noted that most miRNAs identified here have not been previously reported as being differentially expressed in ovarian cancer, and thus, may represent novel diagnostic or prognostic biomarkers.

Details on the exosomal miRNA expression in ovarian cancer cells of different histological subtypes are shown in Table S1. CCCO and endometrioid cancer of the ovary develop from the basis of endometriosis [42,43,44]. In this study, we observed that miR-1281 was upregulated in CCCO cell lines, HAC-2 and OVAS, as well as in the endometrioid cancer cell line, TOV-112D (Table S1), suggesting that this miRNA may be involved in the development of tumors originating from endometriosis. 

### 2.6. Detection of Exosomal miRNA Highly Expressed in CCCO Compared to Other Histological Subtypes

CCCO is one of the most common histological subtypes of endometriosis-related ovarian cancer, which could be detected at the initial stage (stage I) [42,43]. However, advanced CCCO is resistant to standard chemotherapy and has a very poor prognosis [5]. Therefore, early CCCO detection is important to improve the outcome, and the development of highly sensitive and specific markers for monitoring CCCO progression is essential. 

We detected the common exosomal miRNAs overexpressed in the two CCCO cell lines compared to their expression in other histological subtypes; the expression rates in each cell line are shown in Table 3. 

Among the miRNAs significantly overexpressed in CCCO cells, miR-21-5p is one of the first identified miRNAs and has been extensively studied as a representative molecule [45]. Previous studies have shown that the plasma level of exosomal miR-21-5p could be used as a biomarker for several cancers, including breast [46] and colorectal [47] cancers and gastric cancer in young patients [48]. MiR-21 is significantly upregulated in endometriosis-associated ovarian cancer, which is characterized by decreased expression of PTEN [49], a well-known tumor suppressor whose inactivation is an early event of malignant transformation from endometriosis [50]. However, miR-21 has been reported as a non-neoplastic biomarker [45,51]; therefore, miR-21-5p expression in CCCO should be considered with caution. The association of miR-29a-3P with ovarian cancer has also been reported. Thus, miR-29a-3p-absorbing circular RNA circKRT7 has been shown to upregulate the *COL1A1* gene and promote the proliferation and metastasis of ovarian cancer cells [52]. Wen et al. [53] have reported increased miR-30a-5p levels in urine samples of patients with ovarian serous adenocarcinoma, suggesting that miR-30a-5p could be a promising diagnostic and therapeutic target. Meta-analysis performed by Li et al. [54] indicated that the miR-200 and miR-30 families could be promising prognostic biomarkers of ovarian cancer. Thus, the three exosomal miRNAs that were detected in this study as more differentially expressed in CCCO than in other EOC subtypes, thus have potential as CCCO-specific biomarkers.

Finally, we performed target gene and functional pathway prediction for the identified exosomal miRNAs. Table 4 shows the Kyoto Encyclopedia of Genes and Genomes (KEGG) pathway enrichment analysis of miRNAs using KEGG Orthology-Based Annotation System (KOBAS). Figure S1 shows the pathway maps of miR-21-5P and miR-30d-5P gene targets. There was no pathway for genes targeted by miR-29a-3P.

Figure S1 shows the pathways of miRNA target genes in various cancers; KEGG pathway analysis indicated that miR-21 targeted genes involved in tumor suppression, such as *PTEN*, *TPM1*, *PDCD4*, *MASP1*, and others; however, miR-30 gene targets were not detected.

Most studies on exosomal miRNAs in ovarian cancer have used patient plasma. Moreover, reports on the identification of exosomal miRNAs released by cultured cancer cells are nonexistent. Exosomes in serum can be derived not only from cancer cells but also from blood and vascular endothelial cells as well as from other cell types. In this study, we obtained the profiles of exosomal miRNAs released by cell lines corresponding to different histological subtypes of ovarian cancer. Then, we compared the exosomal miRNAs highly expressed in CCCO cells to those in other histological types. The identified miRNAs could represent potential candidate biomarkers for diagnosing or monitoring the progression of ovarian cancer, including CCCO.

## 3. Materials and Methods

### 3.1. Cell Culture

MCAS and HAC-2 cell lines were obtained from the Japanese Collection of Research Bioresources (JCRB) Cell Bank; TOV-112D cell line was obtained from the American Type Culture Collection (Rockville, MD, USA); and HRA [55] and OVAS [56] cell lines were kindly provided by Professor Yoshihito Yokoyama from Hirosaki University School of Medicine, Hirosaki, Japan. The cells were cultured in RPMI-1640 medium (Thermo Fisher Scientific, Tokyo, Japan) supplemented with 10% fetal bovine serum (FBS) and 1% penicillin-streptomycin (Thermo Fisher Scientific). Normal HOSE cells were purchased from ScienCell Research Laboratories (San Diego, CA, USA) and cultured in ovarian epithelial cell medium supplemented with 1% Ovarian Epithelial Cell Growth Supplement and 1% antibiotic solution (ScienCell). All cell cultures were maintained at 37 °C in a humidified incubator containing 5% CO_2_. 

### 3.2. Exosome Isolation from Culture Media 

Cells were seeded in 100 mm culture dishes and cultured until confluence. To prevent contamination with FBS-derived exosomes, the cells were washed twice with FBS-free medium and cultured in 10 mL of FBS-free medium/dish for 48 h. Then, the culture media were collected from the dish and centrifuged at room temperature (RT; approximately 20 °C) 1750× *g* for 15 min. Next, the supernatant was filtered through a 0.2 μm filter to remove cellular debris, and 10 mL was used for exosome isolation with the ExoEasy Maxi kit (Qiagen, Hilden, Germany) according to the manufacturer’s protocol. In total, 700 μL of intact exosomes were obtained.

### 3.3. Nanoparticle Tracking

NTA of exosomes was performed using Quantum Design Japan (Tokyo, Japan). Exosome concentration and size distribution were analyzed using NanoSight (LM10V-HS; Malvern Panalytical Ltd., Malvern, UK) equipped with NTA software (version 3.1; Malvern Panalytical Ltd., Malverin, UK).

### 3.4. Western Blotting Analysis

Intact exosomes (100 μL) were dried in a Savant SpeedVac (Thermo Fisher Scientific), dissolved in 20 μL of sodium dodecylsulfate (SDS) sample loading buffer (EzApply; ATTO, Tokyo, Japan), and boiled for 5 min at 95 °C. Proteins were separated in 10–20% SDS-PAGE gels (e-PAGEL; ATTO, Tokyo, Japan) and transferred to PVDF membranes (ATTO), which were blocked with PVDF Blocking Reagent for Can Get Signal^®^ (TOYOBO Biotech support Department, Osaka, Japan) for 1 h at room temperature (RT; approximately 20 °C). Immunodetection was performed by incubation with anti-CD63 (H-193) rabbit antibody (dilution 1:600; Santa Cruz Biotechnology, Inc., Santa Cruz, CA, USA) for 60 min at RT and then with horseradish peroxidase-conjugated anti-rabbit IgG (dilution 1:2000; Cell Signaling Technology Japan, Tokyo, Japan) for 60 min at RT. Signals were generated using an ECL kit (GE Healthcare, Tokyo, Japan) according to the manufacturer’s protocol.

### 3.5. Exosomal miRNA Extraction

Total RNA (including miRNA) was isolated from exosomes using the RNeasy micro kit (Qiagen, Tokyo, Japan) according to a modified protocol. Briefly, intact exosomes (700 µL) were thoroughly mixed with the double volume of 100% ethanol by pipetting and loaded into a RNeasy MinElute spin column, which was centrifuged at 8000× *g* for 15 s at RT (approximately 20 °C). The membrane was washed, and 500 µL Buffer RPE was added to the RNeasy MinElute spin column, which was again centrifuged at 8000× *g* for 15 s; the flow-through discarded and the procedure repeated twice. Next, 500 µL of 80% ethanol was added to the column, which was centrifuged at 8000× *g* for 2 min and then at 15,000 rpm for 5 min to dry the membrane. Finally, 14 µL of RNase-free water was added directly to the column membrane, and RNA, including the miRNA was eluted by centrifugation at full speed for 1 min. Qualitative and quantitative analysis of exosomal RNA was conducted in an Agilent 2100 Bioanalyzer (Agilent Technologies, Foster City, CA, USA) using the Agilent RNA 6000 Pico kit (Agilent Technologies) according to the manufacturer’s instructions. About 40–50 μL of total RNA from each sample was used for microarray analysis.

### 3.6. miRNA Expression Profiling Using Microarrays 

Approximately 50 ng of total RNA was used for microarray analysis; total RNA labeling and hybridization were performed at Macrogen Japan (Tokyo, Japan). MiRNA-specific microarray analysis was also performed at Macrogen Japan using the Sure Print G3 Human miRNA microarray21 (Agilent Technologies); signals were quantified using Feature Extraction 11.0 software (Agilent Technologies). The microarray dataset is available at http://www.ncbi.nlm.nih.gov/geo (submission on 3 March 2022), under the accession code GSE197892.

### 3.7. Target Gene Prediction and Pathway Analysis

Target genes of differentially expressed miRNAs were predicted using RNAhybrid ver. 2.1.2 (https://bibiserv.cebitec.uni-bielefeld.de/rnahybrid (accessed on 11 May 2021)) and TargetScan ver. 7.2 (http://www.targetscan.org/vert_72/ (accessed on 11 May 2021)) databases and subjected to Gene Ontology (GO) functional annotation and KEGG pathway enrichment analysis [57] using KOBAS ver3.0 (http://kobas.cbi. pku.edu.cn/ (accessed on 14 May 2021).

## 4. Conclusions

This study presents exosomal miRNA profiles specific for ovarian cancer cells of four histological subtypes. Exosomal miRNAs overexpressed in CCCO should be validated as potential candidate biomarkers of cancer progression. Although we only used cell lines in this study, our findings provide a basis for further evaluation of the identified exosomal miRNAs in clinical samples, which will be carried out in future studies. 

## Figures and Tables

**Table 1:** Concentration and diameter of extracellular vesicles isolated from culture media of ovarian cancer cell lines.

Parameter	HRA	TOV-112D	HAC-2	MCAS
Mean diameter (nm)	121.4 ± 11.2	243.7 ± 9.7	260.4 ± 8.3	239.4 ± 7.1
Mode diameter (nm)	89.1 ± 5.8	190.5 ± 29.3	214.6 ± 14.2	200.6 ± 4.6
Concentration (particles/mL)	3.80 × 10^8^	5.33 × 10^8^	3.24 × 10^9^	1.58 × 10^10^

**Table 2:** List of the 28 exosomal miRNAs significantly overexpressed in all ovarian cancer cell lines.

1	hsa-miR-6767-5p	15	hsa-miR-4653-3p
2	hsa-miR-4313	16	hsa-miR-106b-5p
3	hsa-miR-25-3p	17	hsa-miR-4299
4	hsa-miR-425-3p	18	hsa-miR-4291
5	hsa-miR-6880-3p	19	hsa-miR-4284
6	hsa-miR-4749-3p	20	hsa-miR-6798-5p
7	hsa-miR-5581-5p	21	hsa-miR-4728-5p
8	hsa-miR-494-3p	22	hsa-miR-574-3p
9	hsa-miR-766-3p	23	hsa-miR-4716-3p
10	hsa-miR-4725-5p	24	hsa-miR-371a-5p
11	hsa-miR-1237-3p	25	hsa-miR-4672
12	hsa-miR-93-5p	26	hsa-miR-4656
13	hsa-miR-378i	27	hsa-miR-320d
14	hsa-miR-4713-3p	28	hsa-miR-1181

**Table 3:** Detection of exosomal miRNAs overexpressed in CCCO (expression rate compared to normal cells is shown).

miRNA	HRA	TOV112D	MCAS	HAC2	OVAS
hsa-miR-21-5p	0.17	0.08	0.53	4.53	2.02
hsa-miR-29a-3p	0.64	0.21	0.53	7.93	2.11
hsa-miR-30d-5p	0.78	0.39	0.50	5.20	2.83

**Table 4:** KEGG pathway enrichment analysis of miRNAs performed using KOBAS.

	Term	Database	ID	Input Number	Background Number	*p*-Value
miR-21-5P	MicroRNAs in cancer	KEGG PATHWAY	hsa05206	5	299	0.003
miR-30d-5P	MicroRNAs in cancer	KEGG PATHWAY	hsa05206	3	299	0.048

## Data Availability

The data presented in this study are available in the article.

## Supplementary Materials

The following supporting information can be downloaded at: https://www.mdpi.com/article/10.3390/molecules27123953/s1, Table S1: The details of exosomal miRNAs expressed in ovarian cancer cells of different histological subtypes; Figure S1: Pathways of miRNA target genes in various cancers.
