# Supplementary material for: Exosomal MicroRNA as Biomarkers for Diagnosing or Monitoring the Progression of Ovarian Clear Cell Carcinoma: A Pilot Study
Source: Molecules. 2022 Jun 20;27(12):3953. doi: 10.3390/molecules27123953 (PMC9228939; doi:10.3390/molecules27123953)
Supplement: Supplementary file 1 [file molecules-27-03953-s001.zip › suplemental data_KayoHorie/saplimental Table1_KayoHorie.pdf]

Table S1 : The details of exosomal miRNAs by combination of histological types.

HAC2	HRA	HAC2 HRA	MCAS	HAC2 MCAS
hsa-miR-6508-5p	hsa-miR-6789-5p	hsa-miR-3162-3p	hsa-miR-3162-5p	
hsa-miR-1304-3p	hsa-miR-8063	hsa-miR-1268a	hsa-miR-4793-5p	
hsa-miR-6766-3p	hsa-miR-602	hsa-miR-4767	hsa-miR-135a-3p	
hsa-miR-24-3p	hsa-miR-1224-5p	hsa-miR-6872-3p	hsa-miR-4746-3p	
hsa-miR-4665-3p	hsa-miR-4532	hsa-miR-8485	hsa-miR-6893-5p	
hsa-miR-4436b-5p	hsa-miR-4516	hsa-miR-320b	hsa-miR-6734-5p	
hsa-miR-23a-3p	hsa-miR-4689			
hsa-miR-191-3p				
hsa-miR-4787-3p				
hsa-miR-27a-3p				
hsa-miR-22-3p				
hsa-miR-4728-3p				
hsa-miR-4433a-5p				
hsa-miR-92a-3p				
hsa-miR-6763-3p				
hsa-miR-6797-3p				
hsa-miR-7111-3p				

HRA MCAS	HAC2 HRA MCAS	OVAS	HAC2 OVAS	HRA OVAS	HAC2 HRA OVAS
hsa-miR-6723-5p		hsa-miR-3198	hsa-miR-30d-5p	hsa-miR-6803-5p	hsa-miR-6127
		hsa-miR-3196	hsa-miR-29a-3p		hsa-miR-19a-3p
			hsa-miR-21-5p		hsa-miR-1260a
					hsa-miR-20a-5p
					hsa-miR-4507
					hsa-miR-574-5p
					hsa-miR-30e-5p
					hsa-miR-29b-3p

MCAS OVAS	HAC2 MCAS OVAS	HRA MCAS OVAS	HAC2 HRA MCAS OVAS	TOV112D
hsa-miR-4478	hsa-miR-4721	hsa-miR-3652	hsa-miR-1249-5p	hsa-miR-5787
hsa-miR-6785-5p	hsa-miR-4485-3p	hsa-miR-4465	hsa-miR-1260b	hsa-miR-5703
	hsa-miR-1234-3p	hsa-miR-937-5p	hsa-miR-6826-5p	hsa-miR-3648
		hsa-miR-4443	hsa-miR-5100	hsa-miR-3610
		hsa-miR-4257	hsa-miR-7977	hsa-miR-4499
			hsa-miR-7975	hsa-miR-4476
			hsa-miR-7641	hsa-miR-4417
			hsa-miR-17-5p	hsa-miR-630
			hsa-miR-16-5p	hsa-miR-6807-5p
			hsa-miR-4286	hsa-miR-3940-5p
			hsa-miR-4485-5p	hsa-miR-345-5p
			hsa-miR-6875-5p	hsa-miR-3141
				hsa-miR-6089
				hsa-miR-4270
				hsa-miR-1908-3p
				hsa-miR-4763-3p
				hsa-miR-6768-5p
				hsa-miR-4745-5p
				hsa-miR-188-5p
				hsa-miR-2861
				hsa-miR-320a

HAC2 TOV112D	HRA TOV112D	HAC2 HRA TOV112D	MCAS TOV112D	HAC2 MCAS TOV112D	HRA MCAS TOV112D
hsa-miR-7152-3p	hsa-miR-6724-5p		hsa-miR-575		hsa-miR-5739
hsa-miR-378a-3p			hsa-miR-3646		hsa-miR-629-3p
			hsa-miR-4530		hsa-miR-6765-3p

HAC2 HRA MCAS TOV112D	OVAS TOV112D	HAC2 OVAS TOV112D	HRA OVAS TOV112D	HAC2 HRA OVAS TOV112D	MCAS OVAS TOV112D
	hsa-miR-4739	hsa-miR-1281		hsa-miR-197-3p	hsa-miR-4787-5p
	hsa-miR-6858-5p			hsa-miR-19b-3p	hsa-miR-4515
	hsa-miR-1305				hsa-miR-4634
	hsa-miR-6869-5p				
	hsa-miR-874-3p				

HAC2 MCAS OVAS TOV112D	HRA MCAS OVAS TOV112D	HAC2 HRA MCAS OVAS TOV112D
hsa-miR-940		hsa-miR-6767-5p
		hsa-miR-4313
		hsa-miR-25-3p
		hsa-miR-425-3p
		hsa-miR-6880-3p
		hsa-miR-4749-3p
		hsa-miR-5581-5p
		hsa-miR-494-3p
		hsa-miR-766-3p
		hsa-miR-4725-5p
		hsa-miR-1237-3p
		hsa-miR-93-5p
		hsa-miR-378i
		hsa-miR-4713-3p
		hsa-miR-4653-3p
		hsa-miR-106b-5p
		hsa-miR-4299
		hsa-miR-4291
		hsa-miR-4284
		hsa-miR-6798-5p
		hsa-miR-4728-5p
		hsa-miR-574-3p
		hsa-miR-4716-3p
		hsa-miR-371a-5p
		hsa-miR-4672
		hsa-miR-4656
		hsa-miR-320d
		hsa-miR-1181
