# Supplementary figures and images for: Exosomal MicroRNA as Biomarkers for Diagnosing or Monitoring the Progression of Ovarian Clear Cell Carcinoma: A Pilot Study
Source: Molecules. 2022 Jun 20;27(12):3953. doi: 10.3390/molecules27123953 (PMC9228939; doi:10.3390/molecules27123953)

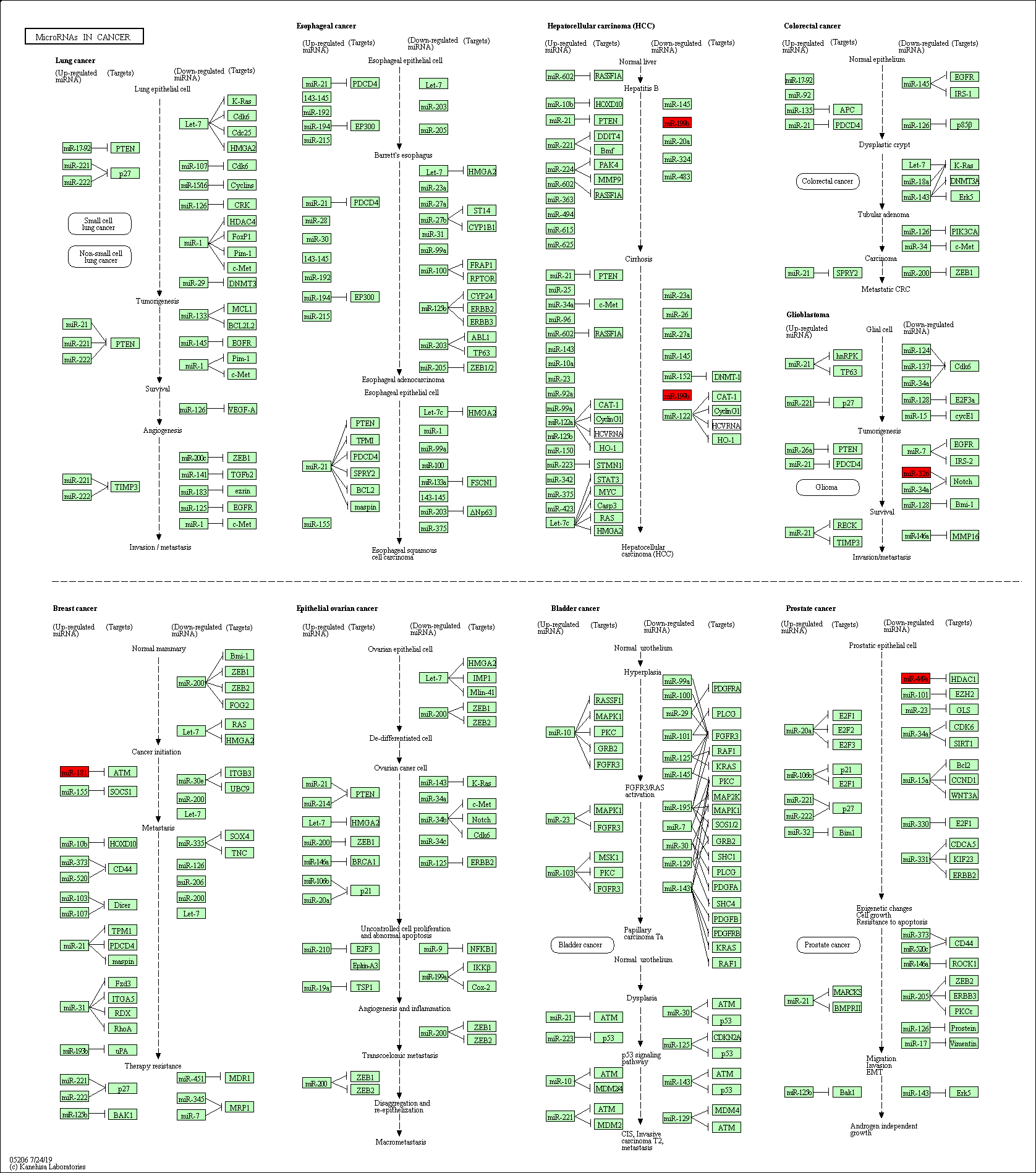

Supplement: Supplementary file 1 [file molecules-27-03953-s001.zip › suplemental data_KayoHorie/saplimental Figure1_ pathways of miRNA target genes_KayoHorie. .png]
